# Secure Base Priming Diminishes Conflict-Based Anger and Anxiety

**DOI:** 10.1371/journal.pone.0162374

**Published:** 2016-09-08

**Authors:** Donald G. Dutton, René A. Lane, Tamara Koren, Kim Bartholomew

**Affiliations:** 1 Department of Psychology, Simon Fraser University, Burnaby, BC, Canada; 2 Department of Psychology, the University of British Columbia, Vancouver, BC, Canada; 3 Faculty of Medicine, Neuroscience, the University of British Columbia, Vancouver, BC, Canada; IRCCS Istituto Auxologico Italiano, ITALY

## Abstract

This study examines the impact of a visual representation of a secure base (i.e. a secure base prime) on attenuating experimentally produced anger and anxiety. Specifically, we examined the assuaging of negative emotions through exposure to an image of a mother-infant embrace or a heterosexual couple embracing. Subjects seated at a computer terminal rated their affect (Pre Affect) using the Affect Adjective Checklist (AAC) then listened to two sets of intense two person conflicts. After the first conflict exposure they rated affect again (Post 1 AAC). Following the second exposure they saw a blank screen (control condition), pictures of everyday objects (distraction condition) or a photo of two people embracing (Secure Base Prime condition). They then reported emotions using the Post 2 AAC. Compared to either control or distraction subjects, Secure Base Prime (SBP) subjects reported significantly less anger and anxiety. These results were then replicated using an internet sample with control, SBP and two new controls: Smiling Man (to control for expression of positive affect) and Cold Mother (an unsmiling mother with infant). The SBP amelioration of anger and anxiety was replicated with the internet sample. No control groups produced this effect, which was generated only by a combination of positive affect in a physically embracing dyad. The results are discussed in terms of attachment theory and research on spreading activation.

## Introduction

Several studies have established connections between attachment insecurity and anger [1–5]. From the perspective of attachment theory [6–9], the original function of anger was to signal separation to an *attachment figure* who is perceived as capable of protecting an infant or child. Separation connotes risk, especially in young, pre-functional and dependent infants, hence, resulting in what Bowlby called an "anger born of fear". The fear is of separation from the base and connotes death. Anger is proposed to be generated by an “attachment behavioural system” that mediates return to proximity, is similar to a stress reaction and including generalized arousal [10]. Whereas anger may be functional when it regenerates proximity to a responsive caretaker, it can become dysfunctional when it leads to alienation or increased socio-emotional distance from an attachment figure. In adulthood, romantic partners are most likely to serve as attachment figures [11, 12] and, correspondingly, to be the targets of intimate anger. Thus, intimate anger and partner abuse may arise when individuals perceive that their connection with a partner is threatened, leading to activation of the attachment system and heightened anger directed toward gaining and maintaining proximity with the partner to reduce experimentally produced anger?

Individuals whose history of experiences with caretakers has led them to lack confidence in their attachment figures are most prone to perceiving threats in close relationships and to responding with dysfunctional anger [13]. In adulthood, individual differences in how individuals respond to potential attachment threats and how they regulate their feelings of security in the context of close relationships can be conceptualized in terms of two dimensions: attachment anxiety and avoidance [14, 15]. Attachment anxiety indicates the extent to which individuals are prone to anxiously anticipate being rejected or abandoned by their partners, and avoidance indicates the extent to which individuals are comfortable approaching their partners for support. Insecure attachment, defined as high anxiety and high avoidance, is associated with dysfunctional beliefs and expectations regarding relationship function and outcomes [16, 17]. In contrast, secure attachment is associated with adaptive emotional regulation [17]. Considerable research documents an association between insecure attachment (and especially attachment anxiety) and proneness to intimate anger and abusive behaviour in romantic relationships [for a review, see Bartholomew & Allison [18]]. This association holds for both men and women, for those in both heterosexual and same-sex relationships, and for university, community, and clinical samples. Could the converse of this finding be produced experimentally: would images of secure attachment produce a diminution of anger and anxiety?

### Secure Base priming

Bowlby proposed that individuals could shift in their attachment security over the lifespan as a function of corrective interpersonal experiences, such as relationships with significant others who are supportive in times of stress. Moreover, he saw a primary goal of therapy to be the restoration of deficits caused by early insecure attachment [19], e.g. through relearning of trust with a consistent therapist [20–22]. Attachment security can also be contextually activated or primed, even among individuals with dispositional low security e.g., Baldwin [23]. This activation has been accomplished through a number of methods, including having research subjects (Ss) imagine interactions with specific supportive others or exposing participants to words associated with security [24, 25]. Mikulincer and Shaver [24] for example, found a Secure Base Priming effect on ameliorating negative outgroup perceptions by using the prime words (in Hebrew) of kirva (closenesss), ahava (love), hivuk (hug), ezra (support). In this study the SBP effect was not produced by positive affect control words e.g.simha (happiness) nor by neutral prime words e.g. misrad (office) for everyday objects.

Shaver and Mikulincer [26] reviewed the affect-regulation function of priming security, focusing on the unconscious or automatic aspect of priming, and concluded that priming enhances breadth of mental categorization and the ability to solve problems creatively. They hypothesized that, because attachment-related strategies are designed to regulate distress, priming or cuing of secure cognitions (that is, a sense that attachment figures are supportive and available) enhances flexibility and exploration of potentially effective responses. Anger is generally antithetical to cognitive flexibility [27–29], hence, techniques that increase cognitive flexibility should decrease anger. Dodge et al. [27] found that dispositional insecure attachment was associated with cognitive problem solving deficits in the form of inflexible other-blaming in conflict situations. This blaming orientation was incompatible with cooperative problem solving strategies. Extrapolating from research examining the impact of security priming on responses to threatening out-groups e.g. Mikulincer & Shaver [24], we might also expect that individuals exposed to security primes would judge conflict situations as less personally threatening and show less reactive anger than those not so exposed.

There is other evidence that priming attachment security can have an ameliorative effect on selected outcome variables. For example, Pierce and Lydon [30] found that the priming of proximity-related words increased reliance on support seeking and decreased reliance on self-denigrating strategies when people were coping with stress. In an older line of work not informed by attachment theory, Silverman and colleagues [31] found that repeated tachistoscopic exposure to phrases such as “Mommy and I are one” designed to generate “symbiotic-like gratifications” (p. 1299) may be a useful adjunct to treatment for problems ranging from obesity [32] to delusional thinking in schizophrenics [33]. More recently, Sohlberg and Birgegard [34] found long term mood effects (over several weeks) from subliminal exposure to this same symbiotic message. However, no research to date has examined whether security priming impacts on affective reactions to intimate conflict, although attachment insecurity appears to be related to relationship dysfunction and to dysfunctional anger [2, 35].

The main explanation for semantic, as well as affective, priming has been spreading activation, a mechanism based on network models of memory [36, 37]. Activation has been thought to spread from the prime to the target when the two share an associative link in semantic memory, thereby influencing decisions about targets. Such activation of associated concepts is assumed to occur automatically and without intention for both semantic priming [37] and affective priming [38].

### Anger/Anxiety in Analog Intimate Conflicts

Attachment security predicts affective responses to written anger-related scenarios [39] and, there is evidence that attachment security is related to experiences of intimate anger [40]. Dutton et al. [2] assessed self-reported anger and anxiety using the Affect Adjective Checklist (AAC) [41] in response to audiotaped scenarios of interpersonal conflict [40]. Moreover, when both situational [42] and individual difference [43] predictors of increased affect were examined—they consistently found that exposure to audios of heated conflict produced heightened self-reported anger and anxiety. This effect appears to be related to past associations of expression of these emotions with reactive fear. It has also been shown that physical abuse victimization was positively related to affective reactivity to audio conflicts [43]. Our main hypothesis in the current study is that priming of attachment security, independent of any dispositional security, will attenuate self-reported anger and anxiety responses to conflict scenarios. This hypothesis stems from prior research reviewed above which indicates effects of primed security on various psychological outcomes e.g., Shaver & Mikulincer [26]. We propose to test this hypothesis with both university and internet samples. We will create experimentally produced affect through exposure to audio conflicts and then attempt to diminish it through secure base priming.

## Method

### Ethics

Ethics approval for human participants was provided by the University of British Columbia Research Ethics Board (IRB) in the Department of Psychology. The UBC Department of Psychology IRB reviewed and approved this study. All participants provided written and/or electronic consent. All participants were required to read and approve the electronic/written consent statements before proceeding. No participants were allowed to participate in this study without consent. Automated pre-screening measures were also in place to verify that the participants' ages were above 18 years. No children were enrolled in this study.

### Participants

#### University group

Data was collected from 686 voluntarily participating university students of which 26.4% and 73.6% were male and female respectively. The students ranged in age from 18 to 59 with a mean age of 20.4 (*SD* = 3.6). The ethnicity/race proportions sample were 57.3% Asian, 32.8% Caucasian, 3.8% Middle Eastern, 3.4% other, 1.9% Latin American, 0.4% Aboriginal and 0.4% African North American. The subjects were recruited by campus flyers, online departmental bulletin boards and those participating for departmental course credit.

#### Internet group

The internet sample encompassed participants from the United States. Of the 278 subjects that partook in the online studies, 58.6% were female and 41.4% were male. They ranged in age from 18 to 69 with a mean age of 34.6 (*SD* = 11.2). Ethnicity/race divisions of the group were 78.8% Caucasian, 7.9% African North American, 7.6% Asian, 5.4% Latin American and 0.4% Aboriginal. The states with the highest numbers of respondents were California, New York and Florida representing 15.5, 8.3 and 7.2% of the total respectively. Income of the participants ranged from unemployed (13.7%) to over $100,000 (1.4%) with bimodal peaks at $20,000-$30,000 and $50,000-$75,000 comprising 14.4 and 14.7% of the total respectively. Education ranged from “Some High School” (1.1%) to those a having a “Professional” degree (2.9%). The majority of the participants had either “Some College or University” credits (39.6%) or an undergraduate degree (32.7%). 12.2% had Masters or Doctoral degrees. In short, they represented a much more diverse and heterogeneous sample than the University Group. The participants were recruited through Mechanical Turk.

### Procedures and Measures

#### Scales used

**The Affect Adjective Checklist (AAC):** A reduced version of the 43 item Affect Adjective Checklist (AAC) scale [41, 44] was used to assess the emotional responses of the participants. Similar to the scale used in Dutton and Webb [40]; subjects were presented with sixteen bipolar affect adjective pairs and had to score each item on a nine-point scale where higher scores represent more intense emotional responses. Three composite items were used to assess the participants’ emotional reactions labelled Anger and Anxiety [40, 45]. Each scale was comprised of three bipolar items. Anger was comprised of rating on angry, aggressive and hostile. Anxiety was comprised of tense, nervous and anxious. Subscale-total score correlations for these composite were reported in Dutton and Webb [40] and ranged from +0.84 (anxiety) to +0.76 (anger).

### Study Design and Procedure

#### Study design

**Conflict audio recordings:** Conflict audios, comprised of three umbrella categories, were created: intimate (couple), stranger, and family conflicts. The intimate scenario consisted of either a jealousy issue or an argument over a weekend activity. Stranger conflicts involved circumstances concerning a road rage incident, expired parking meter or parking spot debacles. Finally, family conflicts comprised of arguments involving parent-child or parent-parent interactions. Additionally, in each audio category, there were sub-variations comprising of male vs. female, female vs. male, male vs. male and female vs. female conflicts. Each S heard two conflict scenarios which were counterbalanced with respect to intimacy vs stranger and sex of actors.

The audios were credibly depicted by professional actors in an unscripted manner and recorded digitally. Audio conflicts involving outside scenarios, such as parking lots etc., had suitable background sounds inserted for enhanced authenticity. The actors were instructed to initiate the argument at a low to moderate level and then after a brief period, escalate the dispute by raising their voices, become verbally abusive and emotionally intense. All scenarios were of similar length and quality with respect to the emotional style, verbal content and emotional intensity. The conflict scenarios were selected from pre-testing for effectiveness (ability to generate emotional reactions as measured by the AAC—see above). Twenty pairs of counterbalanced scenarios were used in the study—these involved a variety of the issues described above as well as various combinations of gender of the persons depicted. Pairs of scenarios (conflict1, conflict 2) were used in a counter-balanced design because we initially intended to contrast affective reactions to stranger vs intimate conflict. However, no significant differences in were found between intimacy, stranger and family scenarios in their ability to generate affect (Anger, Anxiety and general AAC scores), hence, for purposes of data analysis all types of conflict are treated as commensurate. In effect, respondents received two exposures to conflict scenarios before differential exposure to SBP or control/distraction images.

#### Secure base prime and distraction images

Images were presented randomly to Ss in each of the experimental conditions. Each S was exposed to two conflicts and then saw only one image. In order to ensure that anger and anxiety were sufficiently elevated over pre-test levels to permit measurable reductions through SBP, we exposed Ss to two conflict scenarios. Control subjects saw a blank screen for 30s after each conflict and were asked to reflect on the conflict. Secure base prime (SBP) images consisted of either a mother with her infant or a heterosexual couple embracing. The distraction images were pictures of either a stapler, table, whistle or a musical note. These were used to test an alternative explanation for Secure Base Prime effects; namely that they served to distract Ss from aversive emotions. The internet sample had additional controls: a picture of a smiling Caucasian male (SM—Smiling Man) to test whether expression of positive emotion produced the same effect as SBP or a picture of a non-smiling mother holding an infant (CM—Cold Mother) to test whether mother-infant photos without positive affect could produce SBP.

#### Digitization and administration of the study

**University Participants:** The entire study, including the modified AAC, was converted for use on computer stations and implemented using the open source software LimeSurvey under the General Public License agreement. The audio conflicts and images were converted to the Adobe Flash Player (Adobe Systems Incorporated, San Jose, CA) format and embedded within the computer delivered studies. Subjects participated in these computer delivered studies under controlled laboratory conditions.

**Fig 1:**
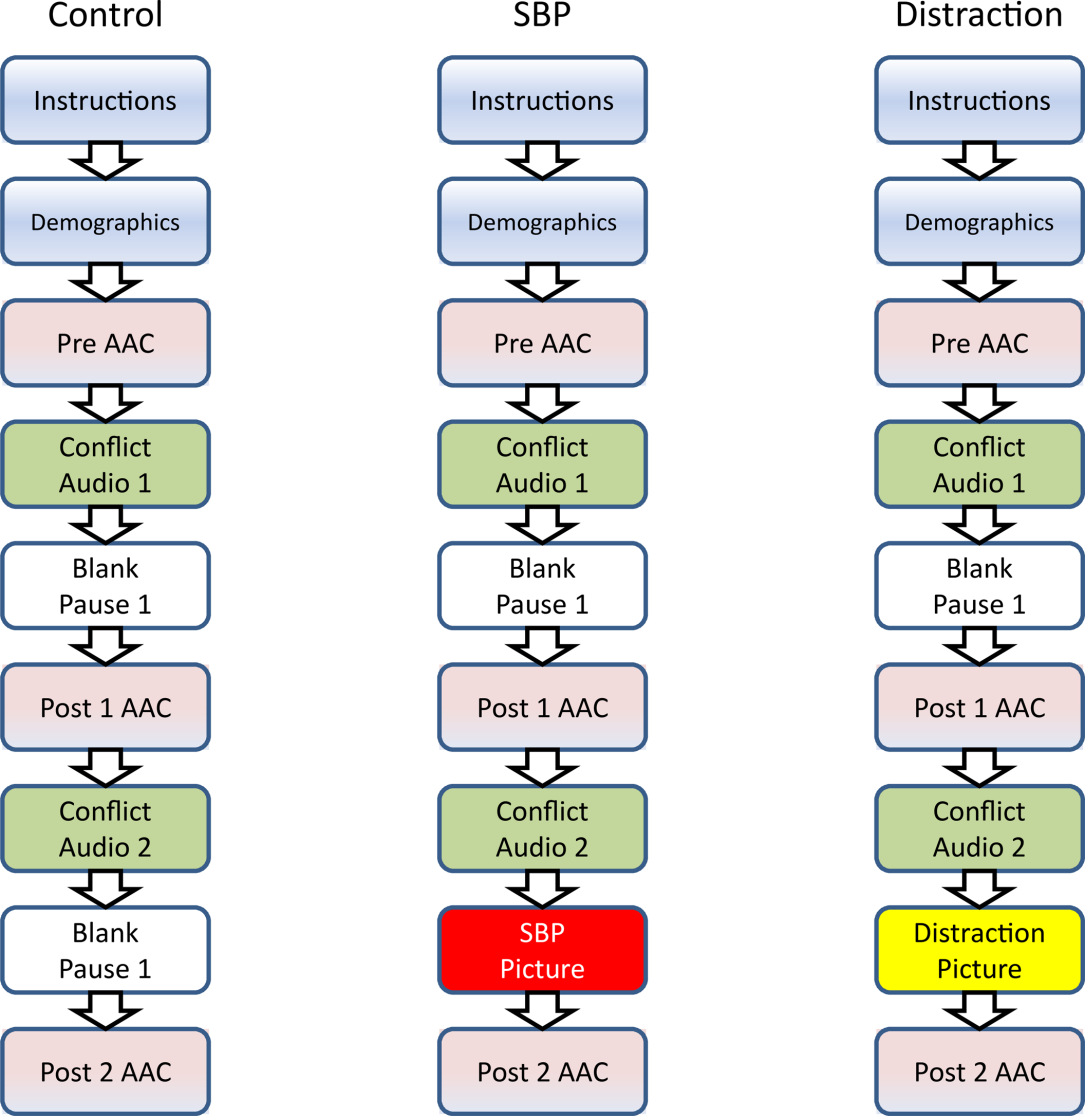
Experimental design and procedural sequence of the original three experimental study groups. Control, SBP (Secure Base Prime), and distraction experiments. Experimental differences between the groups occur after Conflict Audio 2 as indicated by the red (SBP picture) and yellow boxes (distraction picture). AAC = Affective Adjective Checklist (Modified: 16 item).

Participants were instructed to sit in front of computer terminals presented with a full screen view displaying one of the three computer based experiments: a control group without any pictures, the Secure Base Prime picture group or the distraction picture group. As seen in Fig 1, in all the experimental groups, the participants were given instructions on how to complete the study as well as information about anonymity, consent and participation declination at any point during the study. Next, demographic data (gender, socioeconomic status, ethnicity/race) and individual difference assessments (to be presented in a separate paper) were collected. Initial AAC data (Pre AAC) were collected before the first Audio conflict. After the subjects listened to the initial audio conflict, they were instructed to pause for 30s before completing the second AAC (Post1 AAC). They were then presented with the second audio conflict. At this point, the experimental groups differed in the following ways (see Fig 1). The control group received another 30s blank pause, while the SBP group viewed a SBP picture for 30s, and the distraction group viewed a non-human inanimate object picture for 30s. Data for the final AAC (Post2 AAC) were then collected. Our focus will be on Post2 AAC scores in establishing differences amongst groups.

**Internet Participants:** Internet samples are becoming more commonplace in psychological research as internet use becomes normative [46, 47]. We collected the internet portion of our data through the Mechanical Turk (MT) web service. Subjects wanting to participate in our study were pre-screened by reputation (≥ 95% approval rate), adult qualification (≥18 years old), geographical location and prevention of recurrent participation attempts. One advantage of MT over university samples is that the internet sample is generally more heterogeneous [48].

The internet study used the same system that was used with the university sample albeit with some additional safeguards to help ensure uniformity in experience. The university study was conducted under controlled laboratory conditions to ensure that the experience was generally uniform for all subjects. Uniformity was maintained in the internet sample using participant screening, timing controllers on the study itself and pre-participation functionality checks. Anonymity was maintained by using two separate and unlinked databases. The participation experience was visually identical to that of the university group with the exception of additional visual instructions given in lieu of study invigilators reading certain instructions.

#### Calculation of percentage of mean differences

The percentage of mean differences of (Post1 AAC—Pre AAC) and (Post2 AAC—Post1 AAC) were calculated using this formula:

$$AAC\;percentage\;mean\;difference=\left(\frac{\left(AACx-AACy\right)}{8}\right)\times 100\%$$

#### Assessment of valid responses

To check that Ss were attending to the stimuli and assessment questions, subjects were presented with five randomly inserted screening questions throughout the study. The screening questions depicted mostly impossible situations (e.g. “I have swum across a major ocean in the last year” or “a coin was recently minted with my image by the Federal Mint”). Subjects answering these questions could choose from “Strongly Agree (1)” to “Strongly Disagree (4)” on a four-point scale. Those scoring less than 4 on any of the five screening questions were invalidated. Initially, 811 participants’ data were recorded. 15.4% (n = 125) were deemed invalid as per our screening criteria which left 686 valid subjects that were analyzed for this study.

### Statistics

ANOVA was used to determine if there were differences between the experimental groups. *Post hoc* Scheffé and Tamhames tests were used based on the outcome of the Levene statistic. Effect sizes were measured using the Cohen’s d statistic [49]. In all cases significance was set at 0.05.

## Results

### University Group

As with the pre-test, no significant differences were found between conflict scenarios tapping intimacy, family or stranger themes in either the Post1 AAC minus Pre AAC (i.e. Post1 AAC–Pre AAC) or Post2 AAC minus Post1 AAC (i.e. Post2 AAC—Post1 AAC) scores. Since these scenarios had been selected for maximum effectiveness (in terms of generating emotion), this was not surprising.

Post exposure self-report anger and anxiety were calculated by assessing each Post measure's increase in rating above the Pre measure. For the university sample, Table 1 illustrates the mean percentage differences of Post1 AAC—Pre AAC and Post2 AAC—Post1 AAC composite scores. All three experimental groups are similar up to and including exposure to either the initial control or SBP condition; therefore, we would not expect any significant differences in either of the Anger and Anxiety Post1 ACC—Pre ACC scores. Indeed, there were no differences between the groups in the Anger or Anxiety Post1 ACC—Pre ACC scores (Table 1). However, as the experimental conditions changed after Conflict Audio 2, we hypothesized that there should be differences between the SBP and control groups at Post 2 and, indeed this was the case (Table 1). There were significant differences between the SBP and control group in both Anger (*ps* = 0.04) and Anxiety (*ps*<0.001) composites while there were no statistical differences between the distraction and control group in the Post2 ACC Anger (*ps* = 0.97) and Anxiety (*ps* = 0.76) scores.

**Table 1:** Percentage differences between Post1-Pre and Post2-Post1 of the Anger and Anxiety AAC Composite Scores means of the University Group.

ACC Sub Score	Experimental Group	Mean Difference %	*SD*	Sig†
**Anger (Post1 AAC–Pre AAC)**	Control _(n = 222)_	8.09	15.01	0.33
	SBP _(n = 284)_	7.98	15.65	
	Distraction _(n = 180)_	10.02	15.77	
**Anger (Post2 AAC–Post1 AAC)**	Control _(n = 222)_	2.68	14.57	0.01
	SBP _(n = 284)_	-0.51	15.14	
	Distraction _(n = 180)_	3.06	13.14	
**Anxiety (Post1 AAC–Pre AAC)**	Control _(n = 222)_	2.36	19.02	0.51
	SBP _(n = 284)_	2.96	20.19	
	Distraction _(n = 180)_	4.54	17.50	
**Anxiety (Post2 AAC–Post1 AAC)**	Control _(n = 222)_	0.06	14.64	<0.001
	SBP _(n = 284)_	-5.62	15.59	
	Distraction _(n = 180)_	-1.09	15.26	

Table Notes

† ANOVA

**Fig 2:**
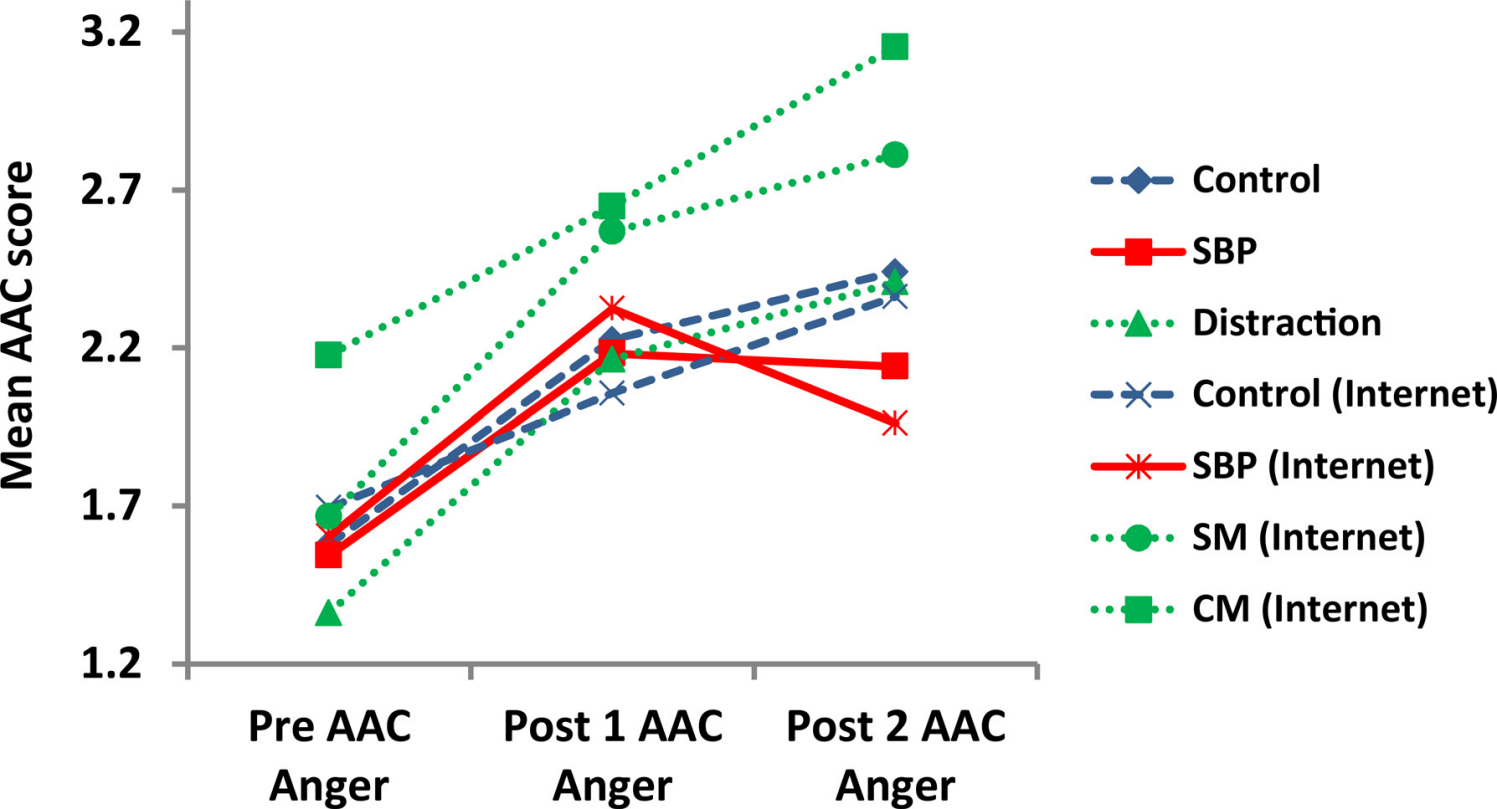
Mean scores for the Anger AAC composite between experimental groups. SBP: Secure Base Prime, SM:”Smiling Man”, CM:”Cold Mother and Child”.

**Fig 3:**
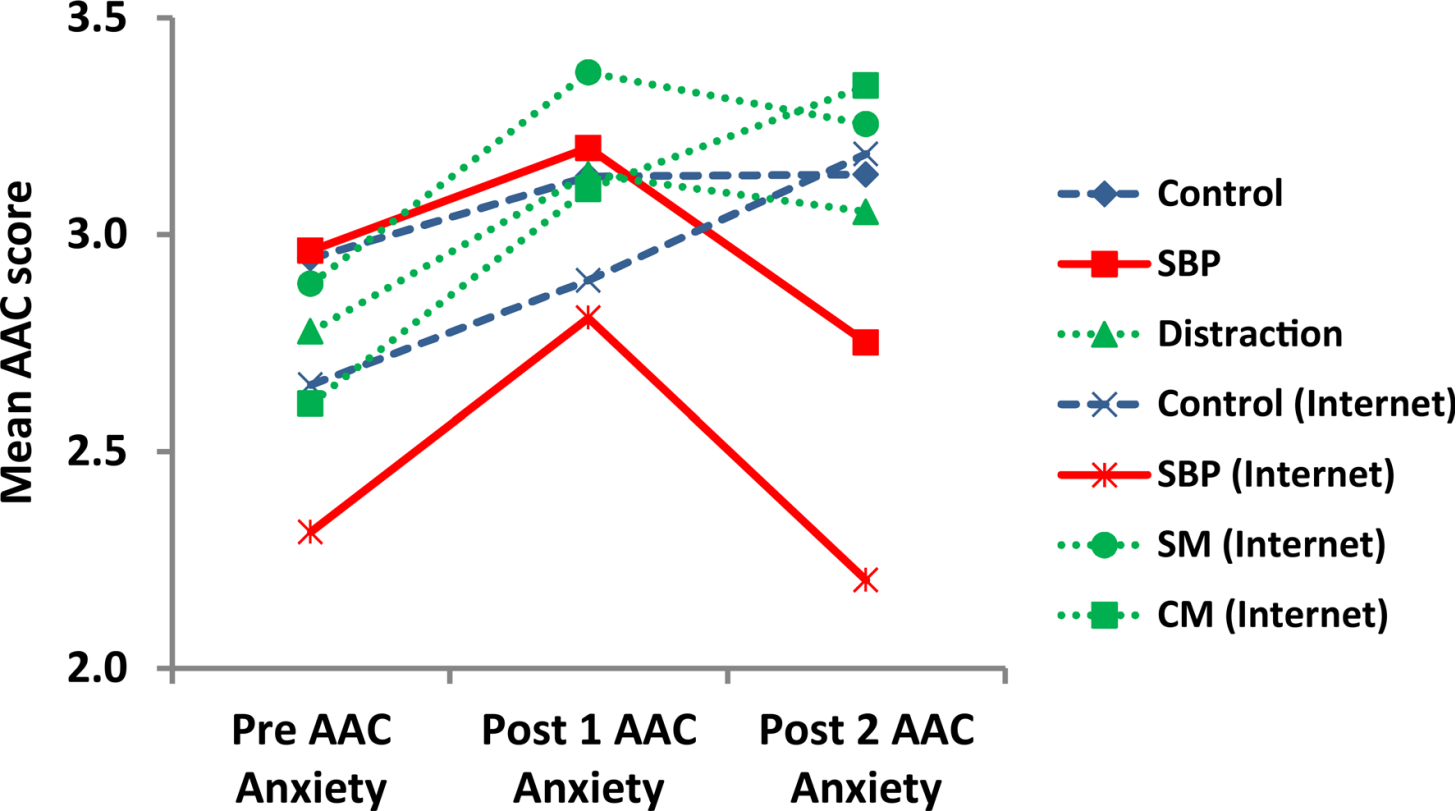
Mean scores for the Anxiety AAC composite between experimental groups. SBP: Secure Base Prime, SM:”Smiling Man”, CM:”Cold Mother and Child”.

Fig 2 and Fig 3 serve to highlight the differences in the Post1 and Post2 ACC Anger and Anxiety scores respectively. As shown in Fig 2 and Fig 3, AAC scores for Anger and Anxiety show similar increasing trends between Pre and Post1 ACC among all groups. However, when the participants have seen the SBP picture, the Anger Post2 ACC—Post1 ACC (Fig 2) score decreases (-0.51%) while the control and the distraction groups show increases in Anger Post2 ACC—Post1 ACC (2.68% and 3.06% respectively) scores. While there is a slight decrease (-1.09%) in the Anxiety Post2 ACC—Post1 ACC score in the distraction group in Fig 3, the decrease is not statistically significant (*ps* = 0.76). Hence, the SBP effect reported above was not due to distraction. Sub-Anger AAC scores (not shown) were similar to anger scores.

#### High scorers–top 20 percent of the university group

Most subjects reported low to moderate affect after hearing the conflict audios. To ascertain whether the SBP effect would occur for stronger levels of emotion, we chose the top 20 percent of subjects in each of the control and Secure Base prime conditions based on their Pre scores on Anger and Anxiety. The highest scorers in the top 20 percent were calculated separately for Anger and Anxiety scores. There were no statistical differences in the Post1 ACC—Pre ACC scores between the SBP and control group in either Anger or Anxiety whereas significant statistical differences were found between the SBP and control group in Post2 ACC—Post1 ACC scores (Table 2). In sum, the top 20 percent highest scoring individuals performed as did the entire university group.

**Table 2:** Percentage differences between Post1-Pre and Post2-Post1 of the Anger and Anxiety AAC Composite Scores means for High Scorers (top 20% of the University Group).

ACC Sub Score	Experimental Group	Mean Difference %	*SD*	Sig
**Anger (Post1 AAC–Pre AAC)**	Control _(n = 60)_	6.88	18.68	0.54
	SBP _(n = 76)_	9.87	23.13	
**Anger (Post2 AAC–Post1 AAC)**	Control _(n = 60)_	2.85	16.02	0.039
	SBP _(n = 76)_	- 4.00	20.40	
**Anxiety (Post1 AAC–Pre AAC)**	Control _(n = 55)_	-7.65	18.94	0.91
	SBP _(n = 75)_	-7.11	24.17	
**Anxiety (Post2 AAC–Post1 AAC)**	Control _(n = 55)_	1.21	13.77	0.001
	SBP _(n = 75)_	- 8.89	17.16	

### Internet Group

We again hypothesized a difference due to SBP in the Post2 ACC—Post1 ACC scores. There were no statistical differences at Post1 ACC—Pre ACC among any of the groups in either Anger or Anxiety (Table 3). However, both Anger and Anxiety Post2 ACC—Post1 ACC scores differed significantly while control and SBP Post1 ACC—Pre ACC scores were not statistically significant (*post hoc* Tamhames *p* = 0.50). Similarly, Anxiety Post1 ACC—Pre ACC control and SBP groups’ scores indicated no statistical differences (Scheffé *ps* = 0.88). Anger and Anxiety Post2 ACC—Post1 ACC scores were strongly contrasted between the SBP and control group as hypothesized. Anger Post2 ACC—Post1 ACC scores increased in the control group by 3.82% while it decreased in the SBP group by 4.53% (Table 3) and was found to be statistically relevant (*ps* = 0.02). Anxiety Post2 ACC—Post1 ACC scores increased by 3.65% in the control group and decreased in the SBP group by 7.55% (Table 3). This too was significant (*ps* = 0.002).

**Table 3:** Percentage differences between Post1-Pre and Post2-Post1 of the Anger and Anxiety AAC Composite Scores means of the Internet Group.

ACC Sub Score	Experimental Group	Mean Difference %	*SD*	Sig†
**Anger (Post1 AAC–Pre AAC)**	Control _(n = 72)_	4.51	15.87	0.12
	SBP _(n = 69)_	8.99	17.19	
	Smiling Man (SM) _(n = 67)_	11.26	23.14	
	Cold Mother (CM) _(n = 70)_	5.89	15.87	
**Anger (Post2 AAC–Post1 AAC)**	Control _(n = 72)_	3.82	14.51	<0.001
	SBP _(n = 69)_	-4.53	16.64	
	Smiling man (SM) _(n = 67)_	3.05	17.29	
	Cold Mother (CM) _(n = 70)_	6.31	14.18	
**Anxiety (Post1 AAC–Pre AAC)**	Control _(n = 72)_	3.01	22.53	0.79
	SBP _(n = 69)_	6.16	22.07	
	Smiling Man _(n = 67)_	6.09	28.12	
	Cold Mother (CM) _(n = 70)_	6.19	16.01	
**Anxiety (Post2 AAC–Post1 AAC)**	Control _(n = 72)_	3.65	19.27	<0.001
	SBP _(n = 69)_	-7.55	17.50	
	Smiling Man (SM) _(n = 67)_	-1.49	19.10	
	Cold Mother (CM) _(n = 70)_	2.98	11.44	

Table Notes

† = ANOVA

Two control groups were added to the repertoire in the Internet Group. The first additional distraction control was an image of a partially “Smiling Man” (SM) and the second was that of an affectively flat woman with a young child or “Cold Mother” (CM). These images were placed in the same experimental position as the SBP image. We again hypothesized that there would be no differences between the groups in the Anger and Anxiety Post1 ACC—Pre ACC differences and this was indeed the case (Table 3). There were, however, significant differences in conditions across the four groups in Anger and Anxiety Post2 ACC—Post1 ACC scores. Anger Post2 ACC—Post1 ACC scores increased for all the control groups except the SBP group which decreased by 4.53% (Table 3). This difference reached significance for the SBP group (*ps* = 0.02) but not for the distraction (SM) or (CM) groups (*ps* = 0.99 and *ps* = 0.83 respectively). Anxiety Post2 ACC—Post1 ACC scores also increased for the control and distraction (CM) group, but decreased slightly by 1.49% for the distraction (CM) and significantly for the SBP group by 7.55% (Table 3). *Post hoc* analysis (Scheffé) indicates that despite the slight reduction in the Anxiety Post2 ACC—Post1 ACC score for the distraction (SM) group, it was found not to be statistically significant to the control group (*ps* = 0.38). The other distraction (CM) group did not reach statistical significance either (*ps* = 0.99) whereas the SBP group did reach significance for Anxiety Post2 ACC—Post1 ACC (*ps* = 0.002).

A general examination of Fig 2 and Fig 3 indicates the general trends of the Anger and Anxiety scores in the Post1 ACC—Pre ACC and Post2 ACC—Post1 ACC conditions. All control groups show increases in scores up to the Post1 ACC point and, similarly, continue to increase in the Post2 ACC point with some minor exceptions. In contradistinction, the SBP decreased in both the Anger and Anxiety Post2 ACC—Post1 ACC scores.

## Discussion

In this study, we increased self-reported anger and anxiety by exposing subjects to audio recordings of angry interpersonal conflicts. We then tested whether subsequent exposure to a secure base prime (a picture of two people embracing) would ameliorate both anger and anxiety. Introduction of a secure base prime did significantly reduce anger and anxiety responses of subjects exposed to the prime compared to responses of subjects in the control (no image) and or other control conditions (Smiling Man, Cold Mother) as indicated by significant between group differences in Post 2 AAC—Post 1 AAC Anger and Anxiety scores. This effect was not simply produced in minimal anger/anxiety conditions; it was replicated with High Scorer groups. It was also replicated with an internet sample. It was not due to distraction. There were no significant differences in Post 2 AAC—Post 1 AAC Anger and Anxiety scores between distraction and control condition subjects. It was produced by an image of a mother and infant embracing or by a heterosexual couple embracing. In each image, positive emotion is displayed. Control pictures used for distraction and/or positive emotion did not produce a SBP effect. An image showing positive emotion *per se*, did not produce the SBP effect, nor did an image of an emotionless mother holding her infant. The effect was only produced by images of a dyadic embrace where positive emotion was expressed.

A number of emotion inducing still photos have been used in emotion research [50] and rated on dimensions of pleasure and arousal, resulting in the International Affective Picture rating System (IAPS). These intersecting continua are referred to as affective space. Research using the IAPS has found that pictures of erotica and adventure generate the most pleasure and arousal in men. In women, pictures of babies and nature generate the most pleasure. Pictures of animal or human threats generate the most arousal in women. The IAPS photo array does not contain pictures depicting attachment.

We were interested in self-reported ratings of specific emotions rather than the two dimensions of pleasure and arousal studied by the IPPS studies, hence, we used a self-report scale similar to that used by Rottenberg et al. [51]. These authors examined emotional reactions generated by commercial films and argue that the ecological validity generated by this research approach is superior to photo presentation. It has been shown that both video and audio clips of conflicts generated anger and anxiety in past experiments [40, 43, 45] and concurred with Rottenberg et al. [51] that this technique is effective at producing emotion. We note that, in the present study, approximately 10 min exposure to two audio conflicts increased anger and anxiety significantly (Cohen's d = .46-.60 for anger, .10-.24 for anxiety) and that a 30 second exposure to a SBP picture then reduced both emotions significantly (Cohen's d = .16-.46 for anger and .25-.47 for anxiety). In a future study, we would assess the effect of increasing the “dosage” level of the SBP exposure.

Mikulincer and Shaver [24] used Hebrew words for everyday objects such as misrad (office), shulhan (table), sira (boat) and imuna (picture) in their control condition; words chosen specifically because of their neutrality to attachment. Our distraction images (stapler, table, whistle or a musical note) were chosen on the same basis. However, since one could argue that in the visual form, these quotidian objects may not distract, we used the Smiling Man control to control for both distraction and for positive emotions. One could also argue that what generated the SBP effect was not the attachment bond portrayed but rather the positive emotions portrayed in the SBP images. However, our Smiling Man control did not produce an SBP effect. Also an image of an unsmiling mother (Cold Mother) and infant did not produce a SBP effect. It appears that a combination of a dyadic interaction with positive affect is required to produce a SBP effect.

The basis of the SBP effect in attachment theory is as follows: The attachment behavioural system responds to sources of threat with actions designed to generate proximity to a secure base [21]. This is an innate behavioural system that serves an evolutionary function and hence, is "hard wired" (op. cit., p. 10). The process of attachment to a secure base, such as (but not limited to) a mother, provides mental representations in adults. These mental representations (called "*working models*" op. cit., p. 23) are of feelings and images connected to a secure base and serve a process of emotional self-regulation, i.e. they allow the person to self soothe [52]. Attachment "working models" consist of episodic memories of interaction sequences, knowledge of the partner's responses and efficacy of the individual's actions. Like other mental representations, working models are presumably underlain by neural circuits or networks which form excitatory and inhibitory associations with one another (op, cit. p. 24). Typically, semantic stimuli have been used in previous research e.g. Mikulincer & Shaver [24]. In the current study, we presume that our conflict audios serve as a threat source, perhaps because of prior associations with family discord. A prior study using a method similar to the one in this study found that previous exposure to abuse in the family of origin was significantly correlated with heightened responses of anger and anxiety to audio conflicts [43]. In the Jack et al. [43] study, simple anticipation of audiotaped conflicts produced heightened affective responding in subjects with previous family exposure to abuse.

Klauer and Musch [36] reviewed a number of proposed mechanisms for "affective priming", one of which was an evaluative system that was triggered by "the mere presence of an object in one's field of perception" (op.cit, p. 7–8). In their research, affective priming refers to the facilitated processing of an affectively polarized target word when it is preceded by an affectively consistent prime word (e.g. sunshine, love). The dependent measure in these studies is time required to process the target word. This effect occurs whether the words are visually presented or the subject memorizes them and recites them aloud. The typical paradigm in this research pairs the prime and target words in temporal proximity similar to the proximity used in the present study (where the secure base prime was presented immediately after completion of conflict). Klauer and Musch [36], in reviewing affective priming studies, found evidence that priming effects existed for up to two days between prime and target exposure. Experiments that masked the prime e.g. Greenwald, Klinger, and Liu [53] and found masked priming effects support the automaticity of the priming process. Klauer and Musch [36] also review the notion that activation spreads in a neural network through stimulation of one node in that network by exposure to the prime. Virtually all the prior research on affective priming has used verbal primes not images and the extant research issues in that field are better tested by words and semantic processing studies. Pictures are encoded differently than words [54]. PET scan studies indicate that encoding of words results in greater activity in prefrontal and temporoparietal regions associated with language function. Encoding of pictures results in greater activity of bilateral visual and medial temporal cortices. Grady et al [54] suggest that the superior overall memory for pictures may be mediated by automatic engagement of areas important for visual memory, such as the medial temporal cortex. A spreading activation mechanism may also be triggered by images, in the present case secure base images.

This study used subliminal primes of threat related words or neutral words. The threat primes lead to faster identification of subsequent proximity related words than did the neutral prime. The effect was realized only on proximity related words. As Shaver and Mikulincer [26] put it: "a symbolic threat unconsciously activated attachment-related words and automatically biased information processing in a lexical decision task" (p. 30–31). This finding was replicated when the dependant variable was retrieval of names of attachment figures but not other persons. The authors concluded that these results "indicated heightened activation of representations of attachment figures under threatening conditions…the priming of threat words increased accessibility of representations of attachment figures but had no effect on representations of other persons" (p. 31). This effect was greater for securely attached individuals and was specific to proximity-related words. It did not speed up access to words connoting separation and rejection. The authors inferred that attachment had pre-conscious aspects indicated by this specific automaticity and supported a psychodynamic approach to personality. The psychodynamic aspect of attachment and attachment imagery was foreshadowed by the work of both Bowlby and Otto Rank [55]. Both saw attachment-separation as a fundamental motive and lifelong theme.

### Internet Samples

In this study we replicated the SBP effect, found initially in a university sample, with an internet sample. Furthermore, we did not merely use the internet sample to assess opinions or attitudes but as an experimental sample with random allocation of subjects to conditions, all run over the internet. Subjects were presented with the same stimuli and assessed with the same measures in both the university and Internet samples. The effects in terms of emotion measures and the SBP effect were identical. In our view, online experiments present a viable alternative to conventional reliance on university student samples and has an advantage in providing a more demographically heterogeneous sample, thus increasing ecological validity.

### Limitations of the Present Studies

One could raise a question as to whether demand characteristics were present in the current studies—i.e. were the Ss cued to respond by the SBP picture or the control pictures? However, the studies were a between-subject design and the participants had only information about the two conflicts they had heard and the subsequent picture post conflict 2. They were asked a battery of “perceptions of conflict” questions (e.g. “Who started the conflict? Who escalated the conflict”?) in addition to the Affect Adjective Checklist. These would have further masked the intent of the study. They had to complete the Affect Adjective Checklist (AAC) with its' 16 descriptors of emotion. It's hard to see how, on the basis of this limited information, they could have adduced which descriptors were the focus of our study (e.g. Why not humiliated, helpless or sad as opposed to anger or anxiety?) but they responded differently on these other descriptors. Furthermore, even on the anger and anxiety measures, the results differed as shown above, more consistent with a veridical response and a subtle difference than a rote response to demand characteristics.

In the current studies we exposed Ss to secure base primes solely in the Post 2 position–after witnessing two conflicts. Future work should examine participants in the Post 1 position and attempt to assess prophylactic effects from SBP. Also, in the current study we examined the SBP effect as a main effect independent of Ss dispositional "attachment style". Future work should examine the interaction of the SBP with attachment style [56]. We found that a 30s SBP ameliorated anger and anxiety although it did not lower anger to Pre AAC conflict exposure levels. Future studies should examine the effects of increased “dosage” of SBP image exposure and whether a SBP effect can be produced subliminally.

In the present study, exposure to intense two-person conflict, led to increases in self-reported emotion of anger and anxiety in both subject samples. Bowlby described the attachment behavioural system as activated by any threat and de-activated by contact with a secure base. From this theoretical perspective, our present conflicts may have generated symbolic threats or else conflict is experienced as threatening and the Secure Base Prime images activated memories of attachment security and hence, reduction of negative emotion. We used an image in this study to ensure a standardized SBP, however, future work could examine whether exposure to a SBP image at the beginning of the study, with instructions to imagine the image later (i.e. after conflict) could produce a SBP effect, acting in effect, as a positive introject for the participant. The present study suggests that therapeutic benefits may accrue from guided visualization of the type of images used in this study; that they could be combined with relaxation and breathing techniques to maximize stress reduction, generate positive affect and reduce emotional turbulence [57].
